# Elevated LDL-C, high blood pressure, and low peak $\dot{V}$ O_2_ associate with platelet mitochondria function in children—The Arkansas Active Kids Study

**DOI:** 10.3389/fmolb.2023.1136975

**Published:** 2023-03-22

**Authors:** Eva C. Diaz, Sean H. Adams, Judith L. Weber, Matthew Cotter, Elisabet Børsheim

**Affiliations:** ^1^ Arkansas Children’s Nutrition Center, Little Rock, AR, United States; ^2^ Arkansas Children’s Research Institute, Little Rock, AR, United States; ^3^ Department of Pediatrics, College of Medicine, University of Arkansas for Medical Sciences, Little Rock, AR, United States; ^4^ Department of Surgery, and Center for Alimentary and Metabolic Science, University of California, Davis, School of Medicine, Sacramento, CA, United States; ^5^ Department of Nursing Science, College of Nursing, University of Arkansas for Medical Sciences, Little Rock, AR, United States

**Keywords:** blood platelets, mitochondria, cardiometabolic risk (factors), aerobic capacity, body composition

## Abstract

**Purpose:** To evaluate the association of platelet (PL) mitochondria respiration with markers of cardiovascular health in children ages 7–10 years.

**Methods:** PL mitochondrial respiration (n = 91) was assessed by high resolution respirometry (HRR): Routine (R) respiration, complex (C) I linked respiration (CI), and maximal uncoupled electron transport capacity of CII (CII_E_) were measured. The respiratory control ratio (RCR) was calculated as the ratio of maximal oxidative phosphorylation capacity of CI and CI leak respiration (P_CI_/L_CI_). Peak $\dot{V}$ O_2_ (incremental bike test) and body composition (dual-energy X-ray absorptiometry) were measured. Multiple generalized linear regression analysis was used to model the association of measures by HRR with variables of interest: adiposity, low-density lipoprotein (LDL-C) and triglyceride (TG) status (normal vs. elevated) HOMA2-IR, blood pressure status (normal vs. high), and demographics.

**Results:** R and CI-linked respiration positively associated with adiposity, high blood pressure (HBP), and peak $\dot{V}$ O_2_. R and CI-linked respiration had inverse association with age and elevated LDL-C. CII_E_ was higher in children with elevated LDL-C (log-β = −0.54, *p* = 0.010). HBP and peak $\dot{V}$ O_2_ interacted in relation to RCR (log-β = −0.01, *p* = 0.028). Specifically, RCR was lowest among children with HBP and low aerobic capacity (i.e., mean peak $\dot{V}$ O_2_ -1SD). HOMA2-IR did not associate with measures of PL mitochondria respiration.

**Conclusion:** In PL, R and CI-linked mitochondrial respiration directly associate with adiposity, peak $\dot{V}$ O_2_ and HBP. Elevated LDL-C associates with lower CI-linked respiration which is compensated by increasing CII respiration. PL bioenergetics phenotypes in children associate with whole-body metabolic health status.

## Introduction

Using platelets (PL) as a minimally-invasive model to study human mitochondria was initially proposed 50 years ago (Salganicoff and Fukami, 1972; Fukami and Salganicoff, 1973). However, technologies capable of measuring mitochondrial function in PL isolated from small volumes of blood are relatively new (Jang et al., 2017), and more research is needed to establish which aspects of PL bioenergetics phenotype associate with a person’s metabolic health or disease risk. To date, studies characterizing PL mitochondrial function in children in states of health and disease remain especially scarce.

PL are anucleate cytoplasmic fragments of megakaryocytes and represent an easily obtainable source of functional mitochondria. While best known for their prominent role in hemostasis, PL are also important mediators of inflammatory and immune processes that contribute to the development of cardiovascular (CVD) and atherosclerosis disease (AD) (Morrell et al., 2014). Low density lipoprotein cholesterol (LDL-C), for instance, upregulates the expression of PL surface molecules and the release of soluble mediators that promote leukocyte recruitment, adhesion to vascular endothelium, and migration into the vascular interstitium (Assinger et al., 2014).

Several studies in adults have indicated that measurements of PL mitochondria respiration could serve as biomarkers of complex systemic diseases (e.g., cancer, rheumatoid arthritis, and neurodegenerative diseases) (Ehinger et al., 2015; Fisar et al., 2019; Rose et al., 2019; Gvozdjakova et al., 2021; Palacka et al., 2021). Others have reported moderate to strong correlations between specific parameters of PL and skeletal muscle mitochondria respiration (e.g., complex I leak respiration, maximal respiratory capacity, and coupling efficiency) (Acin-Perez et al., 2014; Braganza et al., 2019). This points to the possibility that PL could serve—at least to some degree—as a surrogate for skeletal muscle biopsies with respect to determination of mitochondrial bioenergetics. While the quality of evidence in this area remains weak, use of PL mitochondrial bioenergetics phenotypes to understand differences in whole-body metabolic health remains a viable possibility.

How measurements of PL mitochondrial function of children relate to progression of vascular dysfunction and AD needs more investigation. In rats with chemically induced type 1 diabetes, PL p-selectin expression—an adhesion molecule and contributor to AD progression—was upregulated and directly associated with PL mitochondrial maximal electron transport capacity (ETC, r = 0.48) and routine respiration (r = 0.75) (Burger and Wagner, 2003; Siewiera et al., 2016). Importantly, *in vitro* reduction of complex (C) I activity with metformin in PL of rats with a type 2 diabetes-like phenotype decreased p-selectin expression, reactive oxygen species production, and limited thrombosis extent (Xin et al., 2016).

While studies in animal models suggest that PL mitochondria may serve as therapeutic target or as biomarkers to track cardiometabolic health status, the scarcity of human data limits the translatability of these findings. Furthermore, critical knowledge gaps regarding the association of traditional CVD risk factors with PL mitochondria function remain unaddressed. For instance, PL oxidative phosphorylation (OXPHOS) capacity tracked improvements in peak $\dot{V}$ O_2_ and left ventricular ejection fraction in patients with heart failure participating in exercise training (Chou et al., 2019). However, these results have not been recapitulated in other adult populations (Heimler et al., 2022). There is a dearth of studies evaluating the impact of dyslipidemia and systemic high blood pressure on PL mitochondria function. *In vitro* exposure to the saturated fatty acid palmitate results in decreased, ETC in skeletal muscle mitochondria (Yang et al., 2012). Similarly, there is some evidence indicating that PL mitochondria reserve capacity increases in idiopathic pulmonary hypertension (Nguyen et al., 2017), but whether this is true in systemic high blood pressure is not known.

The purpose of this study was to evaluate the association of PL mitochondrial function (i.e., high resolution respirometry) with markers of cardiometabolic health in children. Based on the limited evidence in rodent models and adults, we hypothesized that measures of PL mitochondria respiration positively associate with markers of insulin resistance, adiposity, high blood pressure (HBP), and peak V̇O_2_ while they negatively associate with dyslipidemia in children.

## Methods

### Subjects

A subset of 91 children (7–10 years old) enrolled in the Arkansas Active Kids Study (AAK, NCT03221673) and for which we had access to blood platelets were included in analyses (Table 1). The purpose of the AAK study was to evaluate how physical fitness, physical activity, and obesity status associate with markers of cardiometabolic health in children. Details of the study population and protocols have been described previously (Bai et al., 2020).

**TABLE 1:** Substrate—uncoupler—inhibitor titration (SUIT) reference protocol.

Steps (concentrations)	Measurement	Abbreviation
No additions	Routine respiration	R
Pyruvate and malate (5 mM + 2 mM)	Leak respiration of CI	L_CI_
ADP (2.5 mM)	OXPHOS capacity of CI	P_CI_
Cytochrome c (10 µM)	Outer mitochondrial membrane integrity	Cyt
CCCP (0.5 µM, titrations)	Maximal electron transfer capacity of CI	E_CI_
Glutamate (10 mM)	Replenishing the TCA cycle	G
Succinate (50 mM)	Maximal uncoupled electron transport capacity of CI and CII	E_CI&CII_
Rotenone (0.5 µM)	Maximal uncoupled electron transport capacity of CII: rotenone and succinate	CII_E_
Antimycin A (2.5 µM)	Residual oxygen consumption	ROX
Ascorbate + TMPD (2 mM + 0.5 mM)	Uncoupled CIV electron transport capacity	E_IV_

ADP, adenosine diphosphate; OXPHOS, oxidative phosphorylation; C = complex; CCCP, carbonyl m-chlorophenyl hydrazine (1 µL increments until a maximal plateau was reached); TMPD = N,N,N′,N′-tetramethyl-p–phenylenediamine.

Exclusion criteria were severe persistent asthma (determined by daily use of oral/inhaled corticosteroids to keep asthma symptoms under control and/or frequent use of rescue inhaler), metabolic/endocrine diseases (e.g., type 1 or type 2 diabetes mellitus, hypothyroidism), hormonal replacement therapy, cancer, autoimmune diseases and bleeding disorders. Qualifying children attended a 1-day study visit at the Arkansas Children’s Nutrition Center (ACNC) Laboratory for Active Kids and Families pediatric exercise science facility. The Institutional Review Board (IRB) at the University of Arkansas for Medical Sciences approved the study protocol (IRB #206217). All parents and children gave written informed consent and assent, respectively.

### Anthropometry and body composition

In the overnight-fasted state, body weight and height were measured using a digital scale (Seca 877, Seca GbmH & Co. KG, Hamburg, Germany) to the nearest 0.1 kg and 0.1 cm, respectively, and triplicate values were averaged. Body composition was assessed using dual-energy x-ray absorptiometry (DXA, Horizon-A with Advanced Body Composition™, Hologic, Bedford, MA, United States). Fat mass (FM) index [FMI = FM (kg)/height (m^2^)] and fat-free mass (FFM) index [FFMI = FFM (kg)/height^2^ (m)] z-scores were computed using normative values in children (Weber et al., 2013).

### Blood pressure measurements

Children were asked to empty their bladders and rest lying down for a minimum of 20 min. Blood pressure was measured in duplicate with a 1-min interval on the right arm using an electronic vital sign monitor (CARESCPE™ VC150, Milwaukee, WI, United States). For data analyses, systolic (SBP) and diastolic (DBP) blood pressure percentiles as well as clinical stage were measured; the latter was classified as normal, elevated, stage-1 hypertension, or stage-2 hypertension as per the American Academy of Pediatrics 2017 pediatric blood pressure guidelines (Flynn et al., 2017; Canadian Pediatric Endocrine Group, 2022). In the current study, children with clinical stages of elevated, stage-1 or stage-2 hypertension were considered to have high blood pressure (HBP).

### Cardiorespiratory fitness

Peak $\dot{V}$ O_2_ was assessed through a graded exercise test on a pediatric cycle ergometer (Corival Pediatric, Lode B.V, Groningen, the Netherlands). Oxygen consumption during the exercise test was measured using a metabolic cart (Medgraphics Ultima PFX^®^ system, MGC Diagnostics Corporation, St. Paul, MN, United States). Sitting height was adjusted to a corresponding knee angle of 15° which was measured using a goniometer with the pedal at its lowest position. Crank length was set at 13 cm for 7-year-old children, and 15 cm for 8–10 year-old children (Klimt and Voigt, 1971). The workload increased every minute in increments of 10 W for children <120 cm tall and 15 W for children ≥120 cm tall. During the test, children were instructed to keep the pedal frequency between 50–60 rpm. Children were included for analyses if they met the following criteria during the test: 1) heart rate ≥80% of age-predicted maximum, and/or 2) respiratory exchange ratio ≥1.0, and/or 3) ratings of perceived exertion on the children’s OMNI scale ≥8*.* Careful attention was paid to not terminate the test before children displayed signs consistent with maximal effort. In this study, peak $\dot{V}$ O_2_ is expressed in ml·min^-1^·FFMI^−1^ (Diaz et al., 2021).

### Blood draw and analytes

Blood was drawn between 9:30 a.m. and 10:30 a.m. following an overnight fast *via* venipuncture of the antecubital vein. Blood used for PL isolation (typically, 6 mL) was collected without tourniquet in an EDTA vacutainer and kept at room temperature. Serum concentrations of glucose, triglycerides (TG), and low-density cholesterol (LDL-C), were measured using an RX Daytona clinical analyzer (Randox Laboratories-US Limited, Kearneysville, WV, United States). Lipid status was defined according to the Expert Panel on Integrated Guidelines for Cardiovascular Health and Risk Reduction in Children and Adolescents (i.e., acceptable, borderline, or high) (Expert Panel on Integrated Guidelines for Cardiovascular Health and Risk Reduction in Children and Adolescents. National Heart, 2011). In this study, children with “acceptable” plasma lipid values were considered to have normal status whereas children with either “borderline” or “high” plasma lipids were considered to have elevated lipid status. The threshold point for normal or elevated LDL-C was 2.85 mmol/L whereas the threshold points for TG were 0.85 mmol/L for children 7 to <10 years old and 1.01 mmol/L for children 10 to <11 years of age, respectively (Expert Panel on Integrated Guidelines for Cardiovascular Health and Risk Reduction in Children and Adolescents. National Heart, 2011). Insulin concentration was measured using enzyme-linked immunosorbent assay (Meso Scale Discovery, Rockville, MD, United States). The updated homeostasis model assessment (HOMA2) calculator from the Oxford Centre for Diabetes, Endocrinology and Metabolism was used to estimate insulin resistance (HOMA2-IR) (The Oxford Centre for Diabetes, 2013).

### Platelet mitochondria respiration - high resolution respirometry

#### Platelet isolation

Platelet isolation was done at room temperature and followed recommended isolation protocols for high resolution respirometry (HRR) analysis in platelets (Sumbalova et al., 2017). In brief, whole blood was centrifuged at 200 g for 10 min (acceleration 9 and no brake). Platelet-rich plasma was transferred using a wide bore pipette to a new tube and 10% of a 100 nM EGTA solution added to prevent platelet activation. Platelet-rich plasma treated with EGTA was then centrifuged at 1000 g for 10 min (acceleration 6 and no brake). The PL pellet was gently resuspended in 4 ml of DPBS containing 10 mM EGTA and centrifuged at 1000 g for 5 min. Pelleted PL were resuspended in 0.5 ml of DPBS containing 10 mM EGTA. PL concentration was measured using spectrophotometry, and 200 million cells used for HRR analysis.

#### Simplified overview of oxidative phosphorylation

**FIGURE 1:**
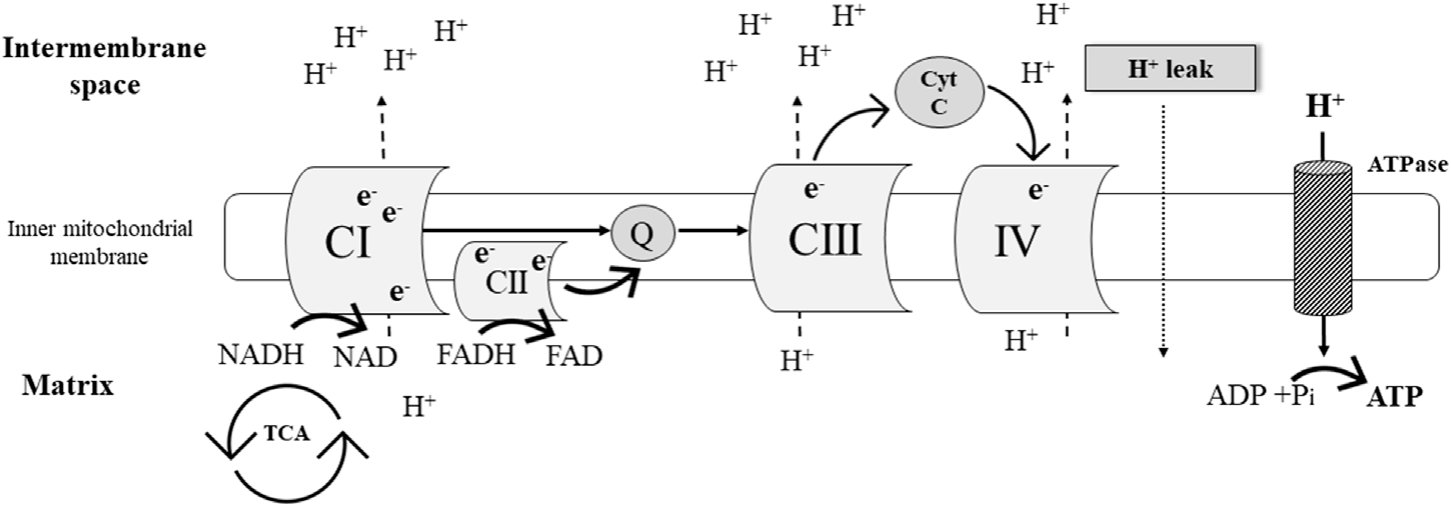
Simplified schematic representation of the mitochondrial electron transport chain.

Oxidative phosphorylation (OXPHOS) is part of the process of aerobic cellular respiration which yields adenosine triphosphate (ATP) as the major energy “currency” in the cell (Figure 1). The OXPHOS system is comprised by chemiosmosis and the electron transport chain. The latter is a collection of four protein complexes and inorganic molecules found in the inner mitochondrial membrane. Electrons [in the form of NADH (Complex I [CI]) and FADH_2_ (complex II [CII])] pass through these complexes in a sequence of redox reactions that enable energy conversions. Most of this energy dissipates as heat, and the rest is utilized to pump hydrogen ions (H+) from the mitochondrial matrix into the intermembrane space (Figure 1). The resulting electrochemical gradient (a.k.a. proton gradient) is used by ATP synthase in chemiosmosis to generate ATP from adenosine diphosphate (ADP) (i.e., ADP phosphorylation) (Ahmad et al., 2021).

#### High-resolution respirometry

Platelet mitochondria respiration (pmol O_2_ s^-1^·10^−6^ platelets) was measured using an O2k-respirometer (Oroboros Instruments, Innsbruck, Austria). Background calibration was performed prior to each experiment as described elsewhere (Rose et al., 2019). To allow the exchange of substrates between the cytosol and respiration medium, PL plasma membranes were selectively permeabilized with digitonin (Gnaiger, 2020). PL mitochondria respiration was evaluated using a substrate-uncoupler-inhibitor titration (SUIT) reference protocol (Table 1).

##### Step 1) Routine respiration

Routine (R) respiration was measured before permeabilization of the plasma membrane. Ten to 15 min were allotted after suspension of PL in the respiration medium for R respiration to achieve a steady state. R respiration is mitochondrial respiration supported by the existing intracellular non-saturating levels of fuel substrates and ADP present in the freshly-isolated PL. R respiration is controlled by cellular energy demands, cellular fuel availability, and the degree by which redox reactions couple to phosphorylation of ADP (Gnaiger, 2020).

##### Step 2) Leak respiration of CI: Pyruvate and malate

Following PL plasma membrane permeabilization, leak respiration of CI (L_CI_) was determined in the absence of exogenous ADP and in the presence of added saturating amounts of NADH-generating substrates [pyruvate and malate]. This results in: 1) the entry and transport of electrons through CI into the Q-junction (i.e., ubiquinone: electron carrier) and further CIII, and CIV; and 2) the pumping of protons (H^+^) from the mitochondrial matrix into the intermembrane space (Figure 1)**.** Because ATP synthase activity is negligible during this process due to limited ADP availability, the electrochemical gradient rapidly increases and drives a flux of protons (H^+^) across the inner mitochondria membrane into the matrix (i.e., proton leak, Figure 1) (Gnaiger, 2020).

##### Step 3) OXPHOS capacity of CI: Saturating concentrations of ADP

Maximal OXPHOS capacity of CI (P_CI_) was induced after the addition of saturating concentrations of ADP and in the presence of NADH-generating substrates pyruvate and malate (Table 1). P_CI_ is influenced by cellular mitochondrial number, mitochondrial surface area, and total numbers of functional electron transport chain components. During P_CI_, the electrochemical gradient generated by H^+^ pumping through CI, CIII, and CIV provides the driving force to support ATP synthase (Gnaiger, 2020) (Figure 1).

##### Step 4. Maximal electron transfer capacity of CI: Protonophore

Maximal electron transfer capacity of (E_CI_) was established with the titration of the protonophore carbonyl cyanide m-chlorophenyl hydrazone (CCCP) (Table 1). CCCP dissipates the electrochemical gradient through induction of unregulated proton leak. During E_CI_, electron transfer across CI remains coupled to H^+^ translocation, but uncoupled to ATP production (Gnaiger, 2020).

##### Step 5. Replenishing the TCA cycle: Glutamate

Glutamate was added to the respiration chamber to ensure a continuous flow of electrons (NADH) into CI. Glutamate is an anaplerotic NADH-linked substrate that undergoes oxidative deamination in the mitochondrial matrix by glutamate dehydrogenase. The products of this reaction are NADH and α-ketoglutarate. In the TCA cycle, the decarboxylation of α-ketoglutarate to succinyl CoA releases NADH as well. After a series of sequential reactions succinyl CoA is converted to malate which is oxidized to oxaloacetate yielding NADH (Gnaiger, 2020).

##### Step 6. Maximal uncoupled CI and CII electron transport capacity: Pyruvate, malate, glutamate, and succinate

Maximal uncoupled CI and CII electron transport capacity (E_CI&CII_) in the continued presence of CCCP was assessed after the addition of succinate (Table 1; Figure 1). Succinate is converted by CII to fumarate yielding the electron donor FADH_2._ In this experimental step, electron transport through CI and CII converges at the Q junction due to the additive effects of CI (malate, pyruvate, and glutamate) and CII (succinate) substrates (Figure 1) (Gnaiger, 2020).

##### Step 7. Maximal uncoupled CII electron transport capacity: Rotenone and succinate

Complex I activity was inhibited with rotenone (Table 1), which allowed the measurement of maximal uncoupled CII electron transport capacity (CII_E_) in the continued presence of CCCP (Gnaiger, 2020).

##### Step 8. Residual oxygen consumption: Antimycin

Residual oxygen consumption (ROX) was measured after CIII was inhibited with antimycin (Table 1) under continued inhibition of CI by rotenone (Gnaiger, 2020).

##### Step 9. Uncoupled CIV electron transport capacity: Ascorbate and tetramethyl-p-phenylenediamine dihydrochloride (TMPD)

Electron flow through CIV (E_IV_) was assessed after the addition of ascorbate and TMPD, respectively. Ascorbate allows TMPD to remain in the reduced state which ensures electron flow through CIV (Gnaiger, 2020).

#### Flux control ratios (FCR)

Oxygen flux in each respiratory state was normalized by E_IV_ (maximal oxygen flux). The estimated FCR allows for internal normalization and expresses respiratory control independent of mitochondria content (Gnaiger, 2020).

#### Respiratory control ratio (RCR)

RCR was calculated as the ratio of maximal OXPHOS capacity (see Step 3, P_CI_) to leak respiration (see Step 2, L_CI_). RCR represents the ability of mitochondria to respond to ADP stimulation, and it is directly associated with substrate oxidation and ATP production (Ojuka et al., 2016).

### Statistical analysis

Data measures in the interval scale are summarized as mean ± SD whereas data measures in the ordinal or nominal scale are summarized as percentages and counts. Categorical variables between groups were compared using the Chi-square or Fisher exact tests.

Data distribution was assessed using the Kolmogorov-Smirnov test. Due to lack of normality in the response variables, a generalized linear model with a natural log link was used to model the association of PL mitochondria respiration with HOMA2-IR, LDL-C status (normal vs. elevated), TG status (normal vs. elevated), blood pressure status (normal vs. HBP), peak V̇O_2_, and subject characteristics (independent variables). Our final models were created using principles of “Purposeful Selection” advocated by Bursac et al. (2008). Briefly, we evaluated several subsets among the important confounding variables, and the best subset of these terms was identified by observation of minimum model Akaike information criterion (AIC) statistic. Statistical analyses were conducted with SAS^®^ 9.4 (Cary, NC, United States).

## Results

### Subject characteristics and metabolic profile

Children (9 ± 1.2 years old) were predominantly Caucasian (73%) (Table 2). Sixty-six percent of children had normal weight and 34% were either overweight or had obesity. One child had missing data for blood analytes. Forty-four children (49%) had elevated LDL cholesterol and 32 (36%) elevated triglyceride (TG) concentrations. Sixty-three (59%) had normal blood pressure and 28 (31%) high blood pressure. Table 3 shows unadjusted flux control ratios at different respiratory states.

**TABLE 2:** Participant characteristics.

Variables	n = 91
Age (years)	9.08 ± 1.24
Sex, n (%)	
Girls	46 (51)
Boys	45 (49)
Race-ethnicity, n (%)	
Black	25 (27)
White	66 (73)
Weight (kg)	33.75 ± 7.95
Height (cm)	135.24 ± 9.02
BMI percentile	65.75 ± 27.49
Weight status, n (%)	
Normal weight	60 (66)
Overweight	13 (14)
Obesity	18 (20)
Fat mass index z-score	0.3 ± 0.69
Fat-free mass index z-score	0 ± 0.85
Insulin (µIU/ml)	1.72 ± 0.63
Glucose (mmol/l)	4.93 ± 0.46
HOMA2-IR	0.87 ± 0.57
Peak V̇O_2_ (ml·min^-1^·FFMI^−1^)	96.42 ± 19.04
Systolic blood pressure percentile	75 ± 20
Diastolic blood pressure percentile	72 ± 18
Blood pressure status, n (%)	
Normal	63 (69)
High	28 (31)
Triglycerides (mmol/l)	0.72 ± 0.28
Triglyceride status, n (%)	
Normal	67 (74)
Elevated	24 (26)
LDL-C (mmol/L)	2.72 ± 0.77
LDL-C status, n (%)	
Normal	46 (51)
Elevated	44 (49)

Data presented as mean ± SD, counts and percentages.

BMI, body mass index, HOMA2-IR, homeostatic model assessment; LDL-C, low density lipoprotein cholesterol.

**TABLE 3:** Unadjusted flux control ratios of permeabilized platelet mitochondria respiration.

Respiratory state	Median	(Q1, Q3)
Routine respiration	0.19	(0.13, 0.25)
Leak respiration of CI	0.15	(0.09, 0.23)
OXPHOS capacity of CI	0.22	(0.16, 0.31)
Maximal electron transfer capacity of CI	0.34	(0.23, 0.46)
Maximal uncoupled electron transport capacity of CI and CII	0.46	(0.26, 0.65)
Maximal uncoupled electron transport capacity of CII	0.06	(0.03, 0.11)
Residual oxygen consumption	0.08	(0.04, 0.16)

Data presented as pmol O_2_·s^-1^·10^−6^ platelets; C = complex; Oxygen flux in each respiratory state was normalized by uncoupled CIV, electron transport capacity (maximal oxygen flux).

### Bivariate associations between subject characteristics and measurements of platelet mitochondrial respiration

FMI z-score (FMIZ) directly associated with R (log-ß = 0.23, *p* = 0.035), L_CI_ (log-ß = 0.34, *p* = 0.029), P_CI_ (log-ß = 0.28, *p* = 0.015), E_CI_ (log-ß = 0.38, *p* = 0.006), E_CI&CII_ (log-ß = 0.33, *p* = 0.008) (Table 4). Similarly, HBP status associated with higher R (log-ß = 0.34, *p* = 0.021), L_CI_ (log-ß = 0.48, *p* = 0.015), P_CI_ (log-ß = 0.37, *p* = 0.015), E_CI_ (log-ß = 0.43, *p* = 0.021), and E_CI&CII_ (log-ß = 0.36, *p* = 0.035). In bivariate analysis, age, sex, HOMA2-IR, elevated LDL-C, elevated TG, and peak V̇O2 did not associate with measurements of PL mitochondria respiration (Table 4).

**TABLE 4:** Bivariate associations between measurements of platelet mitochondrial respiration, metabolic variables, and subject characteristics.

Variables	R		L_CI_		P_CI_		E_CI_		E_CI&CII_		E_CII_		ROX		RCR	
	Log-β	*p*	Log-β	*p*	Log-β	*p*	Log-β	*p*	Log-β	*p*	Log-β	*p*	Log-β	*p*	Log-β	*p*
Age	−0.06	0.297	−0.10	0.213	−0.07	0.279	−0.08	0.326	−0.04	0.602	−0.01	0.893	0.05	0.544	0.04	0.283
Sex	0.23	0.145	0.22	0.306	0.25	0.132	0.22	0.273	0.11	0.519	0.08	0.698	−0.03	0.876	−0.09	0.247
FMIZ	**0.23**	**0.035**	**0.34**	**0.029**	**0.28**	**0.015**	**0.38**	**0.006**	**0.33**	**0.008**	−0.06	0.735	0.01	0.961	0.02	0.756
HBP	**0.34**	**0.025**	**0.48**	**0.015**	**0.37**	**0.015**	**0.43**	**0.021**	**0.36**	**0.035**	0.03	0.895	0.19	0.340	−0.07	0.449
HOMA2-IR	0.08	0.503	0.04	0.822	0.07	0.569	0.11	0.438	0.16	0.187	0.08	0.612	0.07	0.631	0.09	0.192
TG status	0.22	0.761	0.12	0.862	0.19	0.746	0.06	0.852	0.07	0.828	0.01	0.951	−0.19	0.426	−0.1	0.502
LDL-C status	−0.22	0.159	−0.22	0.288	−0.16	0.312	−0.16	0.407	−0.07	0.695	0.39	0.068	0.29	0.143	0.11	0.155
Peak V̇O_2_	0.00	0.393	0.00	0.669	0.00	0.495	0.00	0.467	0.00	0.364	0.00	0.530	0.01	0.202	0.00	0.979

FMIZ, fat mass index z score; SBP, systolic blood pressure percentile; DBP, diastolic blood pressure percentile; HBP, high blood pressure; HOMA2-IR, homeostasis model assessment of insulin resistance; TG, triglycerides (normal status is the reference group); LDL-C, low-density lipoprotein cholesterol (normal status is the reference group). R = routine respiration; L_CI_, leak respiration of CI; P_CI_ = OXPHOS, capacity of CI; E_CI_, maximal electron transfer capacity of CI; E_CI&CII_, maximal uncoupled electron transport capacity of CI, and CII; E_CII_, maximal uncoupled electron transport capacity of CII; ROX, residual oxygen consumption; RCR, respiratory control ratio (P_CI_/L_CI_). Bold values indicate significance below 0.05.

### Generalized multiple linear regression analysis: Final models for measurements of platelet mitochondrial respiration (response variables) and markers of cardiovascular health (independent variables)

**FIGURE 2:**
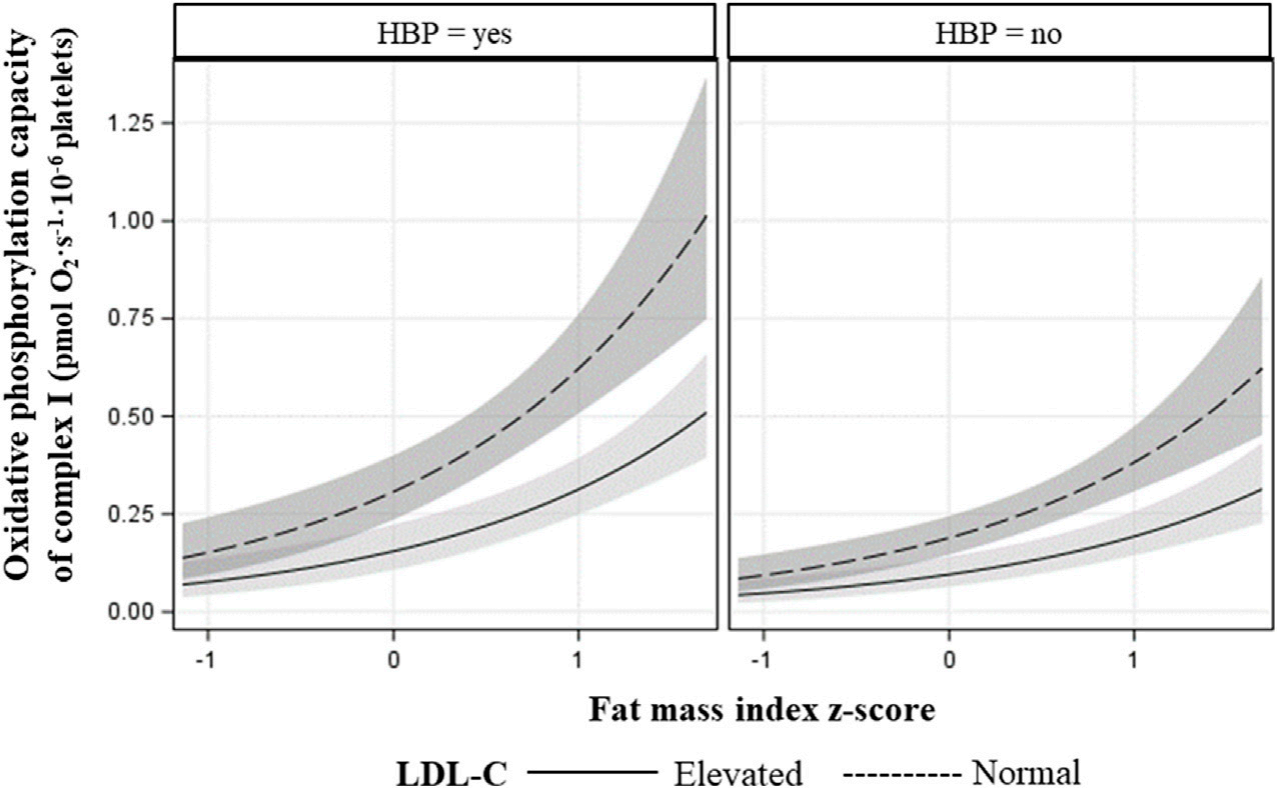
Fitted plot showing the association of CI maximal oxidative phosphorylation capacity plus 95% confidence intervals with fat mass index z-score (x-axis), LDL-cholesterol status, and blood pressure status (HBP = high blood pressure).

The final models for R respiration, CI-linked respiration (L_CI_, P_CI_, and E_CI_), and convergent uncoupled CI and CII respiration (E_CI&CII_), included FMIZ, LDL-C status, HBP, age, and peak V̇O_2_ (Table 5). HOMA-2 IR, sex, and TG status were not retained in any of the final models. Overall, R, CI-linked respiration, and E_CI&CII,_ directly associated with adiposity (*p* < 0.00001), HBP, and peak V̇O_2_ (Tables 5, 6; Figure 2). On the other hand, LDL-C and age were negatively associated with R, CI-linked respiration, and convergent uncoupled CI and CII respiration (Tables 5, 6; Figure 2).

**TABLE 5:** Multiple generalized linear regression analysis modeling the associations of routine and complex I–linked respiration with adiposity, LDL-C status, blood pressure status, age and peak aerobic capacity.

Parameter	R				L_CI_				P_CI_				E_CI_			
	Log-β	CI		*p*-value	Log-β	CI		*p*-value	Log-β	CI		*p*-value	Log-β	CI		*p*-value
FMIZ	0.60	0.35	0.84	<0.0001	1.01	0.71	1.31	<0.0001	0.71	0.46	0.95	<0.0001	1.07	0.76	1.37	<0.0001
LDL-C	−0.66	−0.95	−0.36	<0.0001	−0.92	−1.25	−0.60	<0.0001	−0.69	−0.97	−0.40	<0.0001	−0.89	−1.19	−0.59	<0.0001
Elevated																
Normal (reference)																
Blood pressure status	0.43	0.18	0.69	0.0009	0.63	0.32	0.94	<0.0001	0.49	0.23	0.74	0.0002	0.60	0.30	0.89	<0.0001
High																
Normal (reference)																
Age	−0.19	−0.30	−0.07	0.0018	−0.32	−0.46	−0.18	<0.0001	−0.21	−0.33	−0.09	0.0004	−0.30	−0.44	−0.17	<0.0001
Peak $\dot{V}$ O_2_	0.01	0.00	0.02	0.0191	0.02	0.01	0.02	<0.0001	0.01	0.00	0.02	0.0014	0.02	0.01	0.03	<0.0001

FMIZ, fat mass index z-score, LDL-C, low density lipoprotein cholesterol, R = routine respiration, L_CI_, complex I linked leak respiration, P_CI_, complex I linked oxidative phosphorylation capacity, E_CI_, maximal electron transfer capacity of CI; CI, confidence interval, C = complex.

**TABLE 6:** Multiple Generalized linear regression analysis modeling the association of converged maximal uncoupled electron transport capacity of CI and CII, CII alone, residual oxygen consumption, and respiratory control ratio.

Parameter	E_CI&CII_				E_CII_				ROX				RCR			
	Log-β	CI		*p*-value	Log-β	CI		*p*-value	Log-β	CI		*p*-value	Log-β	CI		*p*-value
FMIZ	0.74	0.43	1.06	<0.0001	−0.26	−0.61	0.09	0.1417	−0.15	0.18	0.77	0.3814	0.04	−0.09	0.16	0.5703
LDL-C																
Elevated	−0.62	−0.96	−0.28	0.0003	0.54	0.09	0.99	0.0100	0.38	0.81	2.99	0.0837				
Normal (reference)																
Blood pressure status																
High	0.49	0.18	0.81	0.0019									−1.02	−1.88	−0.15	0.0211
Normal (reference)																
Age	−0.18	−0.32	−0.05	0.0084												
Peak $\dot{V}$ O_2_	0.01	0.00	0.02	0.0049									0.00	−0.01	0.00	0.2531
HBP x peak $\dot{V}$ O_2_													0.01	0.00	0.02	0.0278

FMIZ, fat mass index z-score, LDL-C, low density lipoprotein cholesterol; C = complex; E_CI&CII_, maximal uncoupled electron transport capacity of CI, and CII; E_CII_, maximal uncoupled electron transport capacity of CII: rotenone and succinate; ROX, residual oxygen consumption; RCR, respiratory control ratio; CI, confidence interval.

The best-fitted model for maximal uncoupled respiration of CII (E_CII_) included FMIZ (log-β = −0.26, *p* = 0.14) and LDL-C status (log-β = 0.54, *p* = 0.0100). Removal of LDL-C status caused a 357% change in the estimate of FMIZ (from log-β = −0.06 to log-β −0.26). Further analyses were conducted using FMIZ and LDL-C as continuous variables (data not shown in tables). Using this approach, FMIZ (log-β = −3.64, *p* = 0.0235) negatively associated with CII_E_ while LDL-C positively associated with E_CII_ (log-β = 0.43, *p* = 0.0017).

**FIGURE 3:**
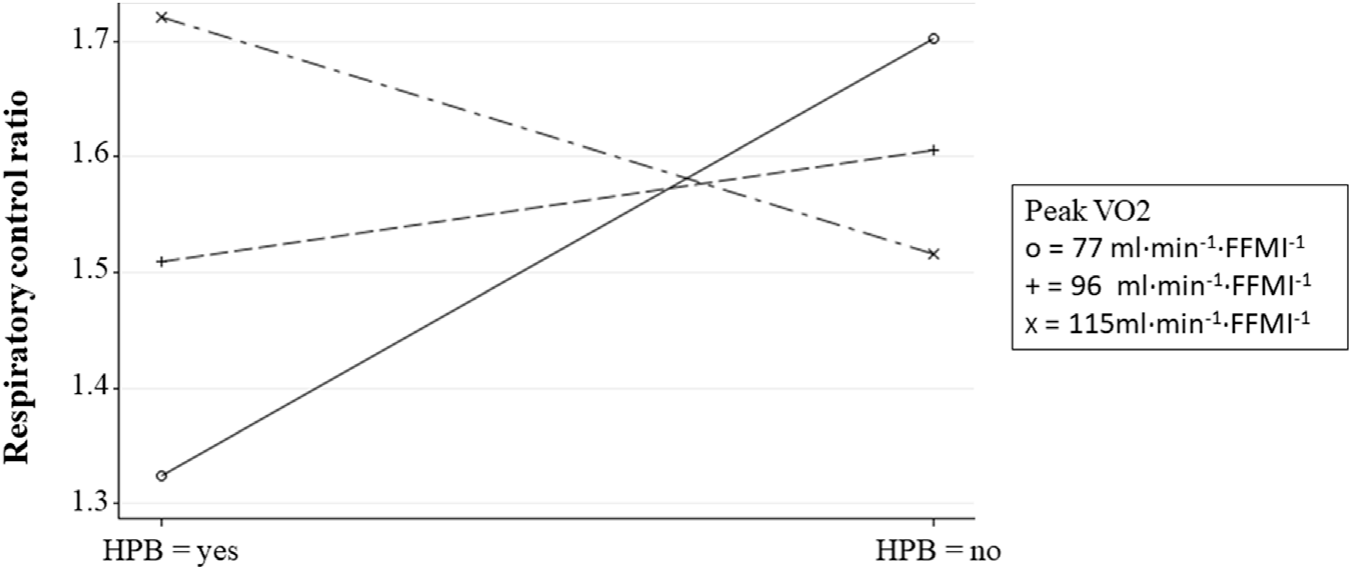
Fitted plot showing the interaction between peak $\dot{V}$ O_2_ and high blood pressure in relation to RCR. Peak $\dot{V}$ O_2_—level groups were created using the group average ± 1SD.

There was a trend (log-β = 0.38, *p* = 0.0837) for elevated LDL-cholesterol to positively associate with ROX. Finally, FMIZ did not associate with RCR. However, there was interaction between HBP and peak V̇O_2_ in relation to RCR (log-β = −0.01, *p* = 0.0278). Specifically, RCR was the lowest among children with HBP and low aerobic capacity (Figure 3).

## Discussion

The purpose of this study was to evaluate the associations of PL mitochondria functions with clinical markers of cardiovascular health of children. A primary finding was that contrary to our hypothesis, HOMA2-IR did not associate with measures of PL mitochondria function. In addition, CI-linked (L_CI_, P_CI_, U_CIE_) respiration positively associated with cardiorespiratory fitness (peak V̇O_2_), adiposity, and HBP while it negatively associated with age. Children with elevated LDL-C had lower CI-linked respiration and higher CII_E_ compared to children with normal LDL-C. Finally, cardiorespiratory fitness modified the association of HBP with RCR. Specifically, children with low aerobic capacity plus HBP exhibited the lowest RCR values.

Elevated mitochondrial membrane potential as well as higher oxygen consumption in the routine, leak, and maximal uncoupled states have been reported in PL of diabetic rats (Siewiera et al., 2016). The authors also reported routine respiration (r = 0.75, *p* < 0.01) ETC (r = 0.48, *p* < 0.01) directly associated with p-selectin expression. The aforementioned study, however, implemented a model of chemically induced type 1 diabetes that is characterized by impaired insulin secretion and not insulin resistance (King, 2012). Therefore, extrapolation of these findings to states of insulin resistance is limited. Using a murine model of type 2 diabetes (high fat diet plus streptozotocin), Xin et al. (Xin et al., 2016) evaluated the effects of metformin (CI inhibitor) on the physiology of ADP activated PL. Mitochondria respiration (routine, leak, and residual oxygen consumption) by HRR was assessed in non-permeabilized ADP-stimulated PL. Compared to metformin-treated rats, PL of non-treated diabetic rats exhibited higher routine respiration while ETS and residual oxygen consumption were not different between groups. The authors reported that inhibition of CI with metformin resulted in downregulation of PL p-selectin expression, lower PL aggregability, decreased reactive oxygen species production, and limited thrombus formation.

The scarcity of literature in this area of research in the adult and pediatric populations limits the ability for comparisons. Our participants were children whose BMI z-scores ranged from normal weight to obesity and who at the time of enrollment were considered healthy. We did not find associations between PL mitochondria function and an indirect measure of insulin resistance (i.e., HOMA-2 IR). Instead, CI-linked respiratory capacity increased in relation to adiposity, and this association did not change when HOMA2-IR was included in the model (data not shown). Our finding that CI linked respiration directly associates with adiposity may reflect the ability of mitochondria to adapt to ATP demands in response to varying degrees of physiological and pathophysiological stimuli (Marchetti et al., 2020). In the present study, flux control ratios were used for analysis to normalize data; therefore, it is unlikely that our finding is the result of differences in mitochondria mass across adiposity levels.

In our study, children with HBP had higher CI-linked (L_CI_, P_CI_, U_CI_) respiration compared to children with normal blood pressure. The lack of data in relation to PL mitochondrial function in patients with systemic HBP is surprising given the prominent role of PL in atherothrombosis and ischemic events in patients with HBP. Measurements of PL bioenergetics by extracellular flux analysis in idiopathic pulmonary hypertension (IPH, i.e., Group 1 pulmonary hypertension) have been reported (Nguyen et al., 2017). Compared to healthy controls, patients with IPH exhibited greater maximal uncoupled respiration and spare respiratory capacity. That said, IPH associated with decreased CI and increased CII protein expression and enzymatic activity, respectively.

We have previously reported that both systolic and diastolic blood pressure percentiles of children improve with increasing peak V̇O_2_, independently of adiposity (Diaz et al., 2021). In addition, the probability of HBP in children with obesity decreased with increasing peak V̇O_2._ Specifically, the probability of HBP in children with obesity decreased by 8% for every unit increase in peak V̇O_2_. In the present study, peak V̇O_2_ was a modifier of the association of HBP and RCR, independently of adiposity. Children with HBP and low peak V̇O_2_ (i.e., mean peak V̇O_2_—1SD) exhibited the lowest RCR values suggesting that low peak V̇O_2_ in children with HBP impairs the ability of PL mitochondria to respond to ADP stimulation, decreases substrate oxidation as well as ATP production (Ojuka et al., 2016). The importance of the associations between fitness, PL mitochondrial dysfunction, and blood pressure in shaping children’s cardiometabolic health status remains to be fully evaluated, and the timing of events remains unknown (i.e., whether or not PL changes follow cardiometabolic phenotypes or *vice versa*). It also remains to be tested if fitness and HBP-associated differences in PL CI and RCR phenotypes recapitulate in metabolically important tissues such as skeletal muscle and liver.

In healthy adults and in patients with heart failure, skeletal muscle mitochondria oxidative phosphorylation capacity is linearly and positively associated with peak V̇O_2_ (Garnier et al., 2005; Chou et al., 2019). Chou et al. (2019) evaluated the effect of high intensity interval training (HIIT) on peak V̇O_2_, cardiac function, and intact PL mitochondria respiration by HRR. The authors found that 12 weeks of HIIT increased left ventricular ejection fraction and that changes in peak V̇O_2_ directly associated with PL ETS (r = 0.79, *p* < 0.0001), and spare respiratory capacity (r = 0.82, *p* < 0.0001). In agreement, our data shows a direct association between peak V̇O_2_ and PL CI-linked respiration, including leak, ETS, and maximal OXPHOS capacity. Taken together, the published evidence and our results suggest that PL mitochondria in children may reflect associations between skeletal muscle mitochondria and peak V̇O_2._ Studies evaluating associations between PL and cardiac muscle mitochondria function in animal models are needed.

A novel finding from this study was that routine and CI-linked respiration decreased in PL of children with elevated LDL-C compared to children with normal LDL-C, independently of adiposity. Our finding is in line with reports of complex interactions between PL and LDL-C (Assinger et al., 2014). LDL-C binds PL *via* Apo-B 100 and modifies plasma membrane lipid composition and fluidity triggering PL activation (Vlasova, 2000). Activated PL release soluble mediators and upregulate the expression of surface molecules that allow PL to interact (indirectly or directly) with immune cells and/or vascular endothelial cells (Siegel-Axel et al., 2008). Furthermore, *in vitro* studies indicate that PL oxidize LDL-C and contribute to the formation of foam cells, which are key players in atherosclerosis initiation (Aviram, 1995). A possible area of future research would be to investigate if the results reported herein reflect PL sensitization to LDL-C.

Interestingly, PL mitochondria of children with elevated LDL-C not only had lower maximal CI-linked respiration but also higher CII uncoupled respiration (CII_E_) compared to children with normal LDL-C. Our finding that CII_E_ has opposite associations to those observed with CI-linked respiration in relation to LDL-C supports the notion that CII activity adjusts to that of CI (Esteitie et al., 2005; Acin-Perez et al., 2014). The latter was identified using skeletal muscle cells of humans with CI deficiency and in mutated mouse cell lines (fibroblasts, T-cells, liver cells) carrying various degrees of CI activity loss (Esteitie et al., 2005; Acin-Perez et al., 2014). It is possible that our findings on CII_E_ reflect an adaptive response to substrate availability (metabolic switch) (Cohen et al., 1970). As an adaptive reaction to stress, cells can consume various metabolic fuels, and depending on the fuel employed, the contribution of electrons derived from NADH and FADH2 varies (Acin-Perez et al., 2014). When fatty acid utilization is favored over glucose utilization, a greater flux of electrons from FADH2 is required. Normally, CIII preferentially accepts electrons from CI; therefore, in order to increase the availability of CIII to accept electrons from CII a reduction in CI activity is needed (Lapuente-Brun et al., 2013).

Our study is limited by its observational design and lack of direct measurements of insulin resistance; however, it is strengthened by the implementation of direct measurements of aerobic capacity and body composition which are frequently missing in studies assessing cardiometabolic risk in children. In summary, an indirect measurement of insulin resistance (i.e., HOMA2-IR) does not associate with PL mitochondria respiration in children. However, CI-linked respiration directly associates with adiposity through unknown mechanisms. LDL-C is negatively associated with CI-linked respiration and positively associated with CII activity. It remains a question if changes associated with higher LDL-C reflect loss of CI function or are in response to greater PL activation and metabolic switch. Combined HBP and low aerobic capacity associate with low RCR, possibly indicating decreased ability of PL mitochondria to respond to ADP to produce ATP. Finally, aerobic capacity directly associates with CI-linked respiration and maximal oxidative phosphorylation capacity of CI. More studies are needed to determine the extent to which variance in mitochondrial phenotypes in children contributes to or reflects the complex interactions of PL with immune and endothelial cells that participate in modifying vascular function, and in the early-life initiation and progression of atherosclerosis.

## Data Availability

The raw data supporting the conclusion of this article will be made available by the authors, without undue reservation.

## References

[B1] Acin-Perez R. Carrascoso I. Baixauli F. Roche-Molina M. Latorre-Pellicer A. Fernandez-Silva P. (2014). ROS-triggered phosphorylation of complex II by Fgr kinase regulates cellular adaptation to fuel use. Cell. Metab. 19, 1020–1033. 24856931 10.1016/j.cmet.2014.04.015 PMC4274740

[B2] Ahmad M. Wolberg A. Kahwaji C. I. (2021). “Biochemistry, electron transport chain [online],” in StatPearls [internet] (Treasure Island, FL: StatPearls Publishing). Available at: https://www.ncbi.nlm.nih.gov/books/NBK526105/ (Accessed Nov 15, 2021). 30252361

[B3] Assinger A. Wang Y. Butler L. M. Hansson G. K. Yan Z. Q. Soderberg-Naucler C. (2014). Apolipoprotein B100 danger-associated signal 1 (ApoBDS-1) triggers platelet activation and boosts platelet-leukocyte proinflammatory responses. Thromb. Haemost. 112, 332–341. 10.1160/TH13-12-1026 24816772

[B4] Aviram M. (1995). LDL-platelet interaction under oxidative stress induces macrophage foam cell formation. Thromb. Haemost. 74, 560–564. 10.1055/s-0038-1642738 8578524

[B5] Bai S. Goudie A. Borsheim E. Weber J. L. (2020). The Arkansas Active Kids Study: Identifying contributing factors to metabolic health and obesity status in prepubertal school-age children. Nutr. Health 27, 273. 10.1177/0260106020975571 33331231

[B6] Braganza A. Corey C. G. Santanasto A. J. Distefano G. Coen P. M. Glynn N. W. (2019). Platelet bioenergetics correlate with muscle energetics and are altered in older adults. JCI Insight 5, e128248. 10.1172/jci.insight.128248 31120438 PMC6629251

[B7] Burger P. C. Wagner D. D. (2003). Platelet P-selectin facilitates atherosclerotic lesion development. Blood 101, 2661–2666. 10.1182/blood-2002-07-2209 12480714

[B8] Bursac Z. Gauss C. H. Williams D. K. Hosmer D. W. (2008). Purposeful selection of variables in logistic regression. Source Code Biol. Med. 3, 17. 10.1186/1751-0473-3-17 19087314 PMC2633005

[B9] Canadian Pediatric Endocrine Group. (2022). R shiny apps from CPEG-GCEP [online]. Available at: https://www.cpeg-gcep.net/ (Accessed Aug 11, 2022).

[B10] Chou C. H. Fu T. C. Tsai H. H. Hsu C. C. Wang C. H. Wang J. S. (2019). High-intensity interval training enhances mitochondrial bioenergetics of platelets in patients with heart failure. Int. J. Cardiol. 274, 214–220. 10.1016/j.ijcard.2018.07.104 30072155

[B11] Cohen P. Derksen A. van Den Bosch H. (1970). Pathways of fatty acid metabolism in human platelets. J. Clin. Invest. 49, 128–139. 10.1172/JCI106211 5409801 PMC322451

[B12] Diaz E. C. Weber J. L. Adams S. H. Young C. G. Bai S. Borsheim E. (2021). Cardiorespiratory fitness associates with blood pressure and metabolic health of children-the Arkansas active Kids study. Med. Sci. Sports Exerc 53, 2225–2232. 10.1249/MSS.0000000000002701 34280939 PMC8516679

[B13] Ehinger J. K. Morota S. Hansson M. J. Paul G. Elmer E. (2015). Mitochondrial dysfunction in blood cells from amyotrophic lateral sclerosis patients. J. Neurol. 262, 1493–1503. 10.1007/s00415-015-7737-0 25893255

[B14] Esteitie N. Hinttala R. Wibom R. Nilsson H. Hance N. Naess K. (2005). Secondary metabolic effects in complex I deficiency. Ann. Neurol. 58, 544–552. 10.1002/ana.20570 16044424

[B15] Expert Panel on Integrated Guidelines for Cardiovascular Health and Risk Reduction in Children and Adolescents National Heart, Lung, and Blood Institute (2011). Expert panel on integrated guidelines for cardiovascular health and risk reduction in children and adolescents: Summary report. Pediatrics 128 (5), S213–S256. 10.1542/peds.2009-2107C 22084329 PMC4536582

[B16] Fisar Z. Hansikova H. Krizova J. Jirak R. Kitzlerova E. Zverova M. (2019). Activities of mitochondrial respiratory chain complexes in platelets of patients with Alzheimer's disease and depressive disorder. Mitochondrion 48, 67–77. 10.1016/j.mito.2019.07.013 31377247

[B17] Flynn J. T. Kaelber D. C. Baker-Smith C. M. Blowey D. Carroll A. E. Daniels S. R. (2017). Clinical practice guideline for screening and management of high blood pressure in children and adolescents. Pediatrics 140, e20171904. 10.1542/peds.2017-1904 28827377

[B18] Fukami M. H. Salganicoff L. (1973). Isolation and properties of human platelet mitochondria. Blood 42, 913–918. 10.1182/blood.v42.6.913.913 4759917

[B19] Garnier A. Fortin D. Zoll J. N'Guessan B. Mettauer B. Lampert E. (2005). Coordinated changes in mitochondrial function and biogenesis in healthy and diseased human skeletal muscle. FASEB J. 19, 43–52. 10.1096/fj.04-2173com 15629894

[B20] Gnaiger E. (2020). Mitochondrial pathways and respiratory control. An introduction to OXPHOS analysis. Bioenerg. Commun. 2020. 10.26124/BEC:2020-0002

[B21] Gvozdjakova A. Sumbalova Z. Kucharska J. Szamosova M. Capova L. Rausova Z. (2021). Platelet mitochondrial respiration and coenzyme Q10 could be used as new diagnostic strategy for mitochondrial dysfunction in rheumatoid diseases. PLoS One 16, e0256135. 10.1371/journal.pone.0256135 34582480 PMC8478238

[B22] Heimler S. R. Phang H. J. Bergstrom J. Mahapatra G. Dozier S. Gnaiger E. (2022). Platelet bioenergetics are associated with resting metabolic rate and exercise capacity in older women [Online]. MitoFit Preprints 2022.7. [Accessed 06-13-2022 2022].

[B23] Jang D. H. Greenwood J. C. Spyres M. B. Eckmann D. M. (2017). Measurement of mitochondrial respiration and motility in acute care: Sepsis, trauma, and poisoning. J. Intensive Care Med. 32, 86–94. 10.1177/0885066616658449 27443317 PMC6902634

[B24] King A. J. (2012). The use of animal models in diabetes research. Br. J. Pharmacol. 166, 877–894. 10.1111/j.1476-5381.2012.01911.x 22352879 PMC3417415

[B25] Klimt F. Voigt G. B. (1971). Investigations on the standardization of ergometry in children. Acta Paediatr. Scand. Suppl. 217, 35–36. 10.1111/j.1651-2227.1971.tb05688.x 5289794

[B26] Lapuente-Brun E. Moreno-Loshuertos R. Acin-Perez R. Latorre-Pellicer A. Colas C. Balsa E. (2013). Supercomplex assembly determines electron flux in the mitochondrial electron transport chain. Science 340, 1567–1570. 10.1126/science.1230381 23812712

[B27] Marchetti P. Fovez Q. Germain N. Khamari R. Kluza J. (2020). Mitochondrial spare respiratory capacity: Mechanisms, regulation, and significance in non-transformed and cancer cells. FASEB J. 34, 13106–13124. 10.1096/fj.202000767R 32808332

[B28] Morrell C. N. Aggrey A. A. Chapman L. M. Modjeski K. L. (2014). Emerging roles for platelets as immune and inflammatory cells. Blood 123, 2759–2767. 10.1182/blood-2013-11-462432 24585776 PMC4007605

[B29] Nguyen Q. L. Corey C. White P. Watson A. Gladwin M. T. Simon M. A. (2017). Platelets from pulmonary hypertension patients show increased mitochondrial reserve capacity. JCI Insight 2, e91415. 10.1172/jci.insight.91415 28289721 PMC5333965

[B30] Ojuka E. Andrew B. Bezuidenhout N. George S. Maarman G. Madlala H. P. (2016). Measurement of beta-oxidation capacity of biological samples by respirometry: A review of principles and substrates. Am. J. Physiol. Endocrinol. Metab. 310, E715–E723. 10.1152/ajpendo.00475.2015 26908505

[B31] Palacka P. Gvozdjakova A. Rausova Z. Kucharska J. Slopovsky J. Obertova J. (2021). Platelet mitochondrial bioenergetics reprogramming in patients with urothelial carcinoma. Int. J. Mol. Sci. 23, 388. 10.3390/ijms23010388 35008814 PMC8745267

[B32] Rose S. Carvalho E. Diaz E. C. Cotter M. Bennuri S. C. Azhar G. (2019). A comparative study of mitochondrial respiration in circulating blood cells and skeletal muscle fibers in women. Am. J. Physiol. Endocrinol. Metab. 317, E503–E512. 10.1152/ajpendo.00084.2019 31211617

[B33] Salganicoff L. Fukami M. H. (1972). Energy metabolism of blood platelets. I. Isolation and properties of platelet mitochondria. Arch. Biochem. Biophys. 153, 726–735. 10.1016/0003-9861(72)90391-8 4662106

[B34] Siegel-Axel D. Daub K. Seizer P. Lindemann S. Gawaz M. (2008). Platelet lipoprotein interplay: Trigger of foam cell formation and driver of atherosclerosis. Cardiovasc Res. 78, 8–17. 10.1093/cvr/cvn015 18218686

[B35] Siewiera K. Kassassir H. Talar M. Wieteska L. Watala C. (2016). Higher mitochondrial potential and elevated mitochondrial respiration are associated with excessive activation of blood platelets in diabetic rats. Life Sci. 148, 293–304. 10.1016/j.lfs.2016.02.030 26872978

[B36] Sumbalova Z. Droescher S. Hiller E. Chang S. Garcia-Valdes L. Calabria E. 2017. O2-Protocols: Isolation of blood cells for HRR [Online]. Available at: http://wiki.oroboros.at/index.php/MiPNet21.17_BloodCellsIsolation (Accessed May 14, 2018).

[B37] The Oxford Centre for Diabetes E. A. M. (2013). HOMA calculator [Online]. Available at: https://www.dtu.ox.ac.uk/homacalculator/download.php (Accessed).

[B38] Vlasova I. I. (2000). The effect of oxidatively modified low-density lipoproteins on platelet aggregability and membrane fluidity. Platelets 11, 406–414. 10.1080/09537100020000157 11132108

[B39] Weber D. R. Moore R. H. Leonard M. B. Zemel B. S. (2013). Fat and lean BMI reference curves in children and adolescents and their utility in identifying excess adiposity compared with BMI and percentage body fat. Am. J. Clin. Nutr. 98, 49–56. 10.3945/ajcn.112.053611 23697708 PMC3683820

[B40] Xin G. Wei Z. Ji C. Zheng H. Gu J. Ma L. (2016). Metformin uniquely prevents thrombosis by inhibiting platelet activation and mtDNA release. Sci. Rep. 6, 36222. 10.1038/srep36222 27805009 PMC5090250

[B41] Yang C. Aye C. C. Li X. Diaz Ramos A. Zorzano A. Mora S. (2012). Mitochondrial dysfunction in insulin resistance: Differential contributions of chronic insulin and saturated fatty acid exposure in muscle cells. Biosci. Rep. 32, 465–478. 10.1042/BSR20120034 22742515 PMC3475448

